# De novo variants of *NR4A2* are associated with neurodevelopmental disorder and epilepsy

**DOI:** 10.1038/s41436-020-0815-4

**Published:** 2020-05-05

**Authors:** Sakshi Singh, Aditi Gupta, Michael Zech, Ashley N. Sigafoos, Karl J. Clark, Yasemin Dincer, Matias Wagner, Jennifer B. Humberson, Sarah Green, Koen van Gassen, Tracy Brandt, Rhonda E. Schnur, Francisca Millan, Yue Si, Volker Mall, Juliane Winkelmann, Ralitza H. Gavrilova, Eric W. Klee, Kendra Engleman, Nicole P. Safina, Rachel Slaugh, Emily M. Bryant, Wen-Hann Tan, Jorge Granadillo, Sunita N. Misra, G. Bradley Schaefer, Shelley Towner, Eva H. Brilstra, Bobby P. C. Koeleman

**Affiliations:** 1 Department of Genetics, University Medical Centre Utrecht, Utrecht, The Netherlands; 2 Center for Individualized Medicine, Mayo Clinic, Rochester, MN USA; 3 Department of Health Sciences Research, Mayo Clinic, Rochester, MN USA; 4 Institut für Neurogenomik, Helmholtz Zentrum München, Munich, Germany; 5 Institut für Humangenetik, Klinikum rechts der Isar, Technische Universität München, Munich, Germany; 6 Department of Biochemistry and Molecular Biology, Mayo Clinic, Rochester, MN USA; 7 Lehrstuhl für Sozialpädiatrie, Technische Universität München, Munich, Germany; 8 Zentrum für Humangenetik und Laboratoriumsdiagnostik (MVZ), Martinsried, Germany; 9 Department of Pediatrics, University of Virginia, Charlottesville, VA USA; 10 Section of Genetics and Metabolism, University of Arkansas for Medical Sciences, Little Rock, AR USA; 11 GeneDx, Gaithersburg, MD USA; 12 kbo-Kinderzentrum München, Munich, Germany; 13 Lehrstuhl für Neurogenetik, Technische Universität München, Munich, Germany; 14 Munich Cluster for Systems Neurology, SyNergy, Munich, Germany; 15 Departments of Clinical Genomics and Neurology, Mayo Clinic, Rochester, MN USA; 16 Department of Neurology, Mayo Clinic, Rochester, MN USA; 17 Division of Clinical Genetics, Children’s Mercy Kansas City, University of Missouri Kansas City School of Medicine, Kansas city, MO USA; 18 Division of Genetics and Genomic Medicine, Department of Pediatrics, Washington University School of Medicine, St. Louis, MO USA; 19 Ann & Robert H. Lurie Children’s Hospital, Epilepsy Center, Chicago, IL USA; 20 Division of Genetics and Genomics, Department of Medicine, Boston Children’s Hospital, Harvard Medical School, Boston, MA USA

**Keywords:** *NR4A2*, epilepsy, seizures, neurodevelopmental disorder, developmental disorder

## Abstract

**Purpose:**

This study characterizes the clinical and genetic features of nine unrelated patients with de novo variants in the *NR4A2* gene.

**Methods:**

Variants were identified and de novo origins were confirmed through trio exome sequencing in all but one patient. Targeted RNA sequencing was performed for one variant to confirm its splicing effect. Independent discoveries were shared through GeneMatcher.

**Results:**

Missense and loss-of-function variants in *NR4A2* were identified in patients from eight unrelated families. One patient carried a larger deletion including adjacent genes. The cases presented with developmental delay, hypotonia (six cases), and epilepsy (six cases). De novo status was confirmed for eight patients. One variant was demonstrated to affect splicing and result in expression of abnormal transcripts likely subject to nonsense-mediated decay.

**Conclusion:**

Our study underscores the importance of *NR4A2* as a disease gene for neurodevelopmental disorders and epilepsy. The identified variants are likely causative of the seizures and additional developmental phenotypes in these patients.

## INTRODUCTION

The *NR4A2* gene encodes a steroid–thyroid hormone–retinoid receptor that acts as a nuclear receptor (NR) transcription factor. The NR transcription factors play a regulatory role in various aspects of mammalian physiology such as neuronal development, inflammation, carcinogenesis, and memory formation. *NR4A2* is required for development, function, and neurotransmission of dopaminergic neurons.^1^

The *NR4A2* protein consists of two main domains: a DNA binding domain (DBD) and a ligand binding domain (LBD). The DBD is a highly conserved domain containing two C4 type zinc fingers that bind to specific motifs in DNA hormone response elements, and is connected to the C-terminal LBD via a linker region. The NR4A2 protein functions by binding small molecule ligands within conserved ligand binding patches located in the hydrophobic core of the LBD.^2^ Ligand binding induces a conformational change in the LBD leading to changes in interaction of nuclear receptor coregulators and other proteins. This alters chromatin structure and gene expression and, therefore, up and down regulation of target genes. A mutated gene may encode for a misfolded protein, dysfunctional ligand binding pocket, or dysfunctional DNA binding.

Haploinsufficiency of the *NR4A2* gene caused by heterozygous chromosomal deletions was previously associated with a neurodevelopmental disorder with high penetrance, suggesting that heterozygous loss of *NR4A2* is autosomal dominant.^3^ *NR4A2* knockout in midbrain dopaminergic neurons of adult mice has shown to result in neuronal degeneration and impaired motor function.^1^ Various polymorphisms in *NR4A2* have been associated with disorders related to dopaminergic dysfunction such as Parkinson disease, schizophrenia, manic depression, and autism spectrum disorder.^4–6^ To our knowledge, there is only one previous report of a variant of *NR4A2* (NM_006186.3:c.327dup, p.S110Vfs*2) associated with epilepsy.^7^

Here we report nine patients with variants in *NR4A2* and developmental delay/intellectual disability with or without epilepsy.

## MATERIALS AND METHODS

Informed consent for genetic testing was obtained from all patients and parents included in the present study. The consent and protocols were approved by the respective institutional ethical review boards (University Medical Centre Utrecht, Mayo Clinic, Children’s Mercy Kansas City, Technical University of Munich, University of Arkansas for Medical Sciences, University of Virginia, Washington University School of Medicine, Boston Children’s Hospital, Ann & Robert H. Lurie Children’s Hospital). All patients underwent exome sequencing; however, the variant identified in patient 2 was detected using a targeted exome analysis for neurodevelopmental genes. The deletion observed in patient 9 was detected by comparative genome hybridization using an Agilent 180K oligoarray. The microdeletion was verified by fluorescence in situ hybridization (FISH). All other cases were analyzed using the complete exome (Supplementary information). By sharing through GeneMatcher,^8^ we discovered other patients harboring variants in the *NR4A2* gene (patients 2, 4, 5, 7). For these patients, exome sequencing was performed at GeneDx (Gaithersburg, MD, USA) using the Clinical Research Exome kit (Agilent Technologies, Santa Clara, CA) or the IDT xGen Exome Research Panel v1.0. The general assertion criteria for variant classification are publicly available on the GeneDx ClinVar submission page (http://www.ncbi.nlm.nih.gov/clinvar/submitters/26957/). All variants identified in patients tested at GeneDx were reported as variants of uncertain significance in accordance with American College of Medical Genetics and Genomics (ACMG) criteria.^9^

**Fig. 1:**
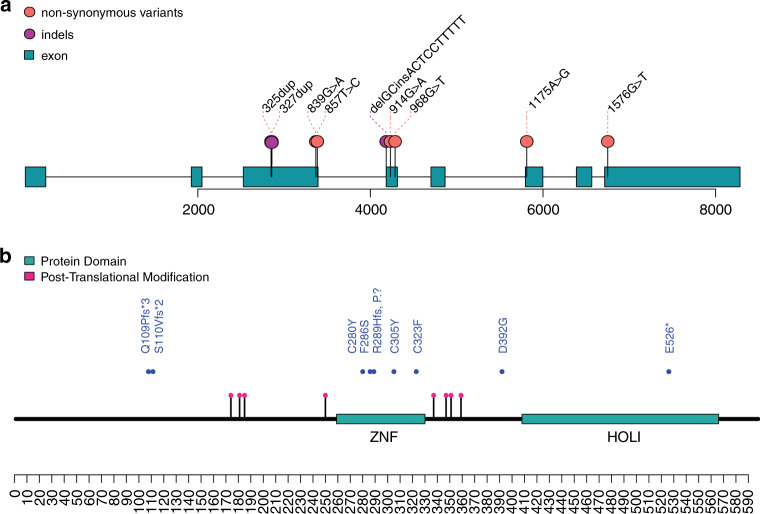
Schematic view of the distribution of pathogenic variants in *NR4A2*. (**a**) Transcript description and locations (marked by circles) of the variants found in *NR4A2* gene. (**b**) Predicted effects (blue dots) of the pathogenic variants on NR4A2 protein sequence. The c.327dup,p.S110Vfs*2 variant was published previously.^7^.

Variants were annotated based on *NR4A2* transcript NM_006186.3. De novo status of the variant was confirmed by exome sequencing or Sanger sequencing of parental samples. In silico tools MaxEntScan, NNSPLICE, and Human Splicing Factor predicted altered splicing and potential loss of function (LoF). Targeted RNA sequencing was done using blood-derived RNA of patient 2 to examine the functional consequences of the variant on splicing. Altered splicing was confirmed by reverse transcription polymerase chain reaction (RT-PCR) (details are in Supplementary Information Figures S1 and S2). Plot Protein and RStudio v1.1.463 were used to visualize the variants in Fig. 1. Predicted domain architecture information for the corresponding amino acids in the protein was retrieved from databases SMART, Prosite, InterPro, and Pfam.

## RESULTS

We report a case series of nine patients with novel variants in *NR4A2*, including eight patients with a confirmed de novo variant, and one patient with a larger deletion encompassing *NR4A2*. The patients (five females, four males; mean age 12.4 years, age range 2–43 years, at the time of inclusion in current study) show heterogeneous phenotypes (detailed phenotypes are given in online Supplementary information). Their neurodevelopmental phenotypes are characterized by delayed psychomotor development (9/9), which was initially normal in two patients. Individuals presented with varying levels (mild to severe) of intellectual disability (ID)/developmental delay (DD). Other features include epilepsy (6/9), speech/language impairment (5/9), behavioral problems (5/9), and movement disorder/hypotonia (8/9). Patients presented with variable epilepsy phenotype, including rolandic epilepsy, generalized encephalopathy, West syndrome, and infantile spasms. One patient showed epileptoform activity and photosensitivity on electroencephalogram (EEG). Seizure type included tonic clonic, generalized, absence, and focal seizures. Seizures remained refractory in two patients and the remaining four became seizure-free on appropriate antiepileptic drugs. Behavioral problems included autism, attention deficit–hyperactivity disorder, hyperactivity, anxiety, and hyposensitivity. Two patients had ataxia. There was no apparent genotype–phenotype correlation.

### Genetic results

We identified eight patients with intragenic *NR4A2* variants and one patients carrying a larger deletion including *NR4A2*. Variants were not present in ExAC and gnomAD. De novo occurrence of the variants was confirmed for eight of these patients. The origin of the variant in patient 5 could not be confirmed due to unavailability of the father, but it was not maternal. Five patients had missense variants, one had a microdeletion, and three had nonsense or frameshift variants leading to a premature stop codon (Table 1, Fig. 1). Four missense variants and one splice-acceptor site variant (c.839G>A, p.C280Y; c.914G>A, p.C305Y; c.857T>C, p.F286S; c.968G>T, p.C323F; c.865-1_865delGCinsAAAAAGGAGT) were located in the DBD of the protein that may affect DNA binding of the transcription factor. Patient 4 carried a missense variant (c.1175A>G, p.D392G) affecting a hinge region without any secondary structure in the LBD. Patient 5 had a nonsense variant (c.1576G>T, p.E526*) in the LBD, introducing premature termination. All missense variants were located in a gene region that is enriched for pathogenic variants across the *NR4A2* gene family (see Supplementary Figure S4).

**Table 1:** Clinical phenotypes of patients with heterozygous de novo and putative de novo *NR4A2* variants.

Patient	Variant (NM_006186.3)	Inheritance	Protein domain/region	Seizures	Age, years/sex/age at seizure onset	Developmental delay	Speech and language impairment	Motor delay	Intellectual disability	Behavioral problems	MRI findings	Neurologic examination findings	Other
1	c.839G>A, p.C280Y	De novo	ZnF_C4 domain, DNA binding domain	Yes	15/F/6.5 years	Global	NA	NA	Severe	Autism	Normal	Normal	Sleeping difficulties
2	c.865-1_865delGCinsAAAAAGGAGT, p.?	De novo	ZnF_C4 domain, DNA binding domain	Yes	12/M/10 years	Global	Yes	Yes	Mild	Hyperactivity, anxiety	Normal	Mild hypotonia	EDS hypermobility
3	c.914G>A, p.C305Y	De novo	ZnF_C4 domain, DNA binding domain	Yes	9/F/NA	Moderate	NA	NA	Mild to moderate	NA	Gliosis	Choreoathetoid movements, dystonia, ataxic gait	NA
4	c.1175A>G, p.D392G	De novo	Hinge region	Yes	3/F/5 months	Global	NA	NA	Severe	No	Moderate cerebellar atrophy	Severe hypotonia, feeding difficulties, dystonia	None
5	c.1576G>T, p.E526*	NA	HOLI, ligand binding domain	No	5/M/Never	Global	Yes	Yes	Mild	Attachment disorder, hyposensitivity	NA	Mild hypotonia, no movement disorder	No
6	c.325dupC, p.Q109Pfs*3	De novo	N-terminal regulatory domain	Yes	2/M/6 months	Global	Yes	Yes	NA	Sensory sensitivity	Pontine hypoplasia, ventriculomegaly	Severe hypotonia, feeding difficulties	Facial dysmorphism, sleep disordered breathing
7	c.857T>C, p.F286S	De novo	ZnF_C4 domain, DNA binding domain	No	4/F/Never	Global	Yes	Yes	Moderate	No	Normal	Hypotonia	Mild joint hypermobility, shagreen spot and hypopigmented spot
8	c.968G>T, p.C323F	De novo	ZnF_C4 domain, DNA binding domain	No	19/F/Never	Global	Yes	Yes	Moderate to severe	No	Normal	Mild generalized hypotonia	Facial dysmorphism, joint hypermobility
9	arr[GRCh37]2q23.3q24.1(154790212_158488241)x1	De novo	NA	yes	43/M/13 years	Moderate	NA	NA	Moderate to severe	Hyperactivity, aggression	Enlarged cerebrospinal fluid spaces	Progressive ataxia in adulthood	No

*AED* antiepileptic drug, *EDS* Ehlers–Danlos syndrome, *EEG* electroencephalogram, *F* female, *M* male, *MRI* magnetic resonance image, *NA* not assessed.

Patient 6 had a frameshift variant (c.325dup) in N-terminal regulatory domain introducing premature termination that is predicted to lead to nonsense-mediated decay (NMD) and LoF, similar to the previously published epilepsy patient (c.327dup).^7^ Patient 9 carried a chromosomal microdeletion arr[GRCh37]2q23.3q24.1(154790212_158488241)x1 of size >3.6 Mb (3698029 bp) encompassing the *NR4A2* gene. The deletion also covered ten flanking genes (*KCNJ3*, *GPD2*, *GALNT5*, *ERMN*, *CYTIP*, *ACVR1C*, *ACVR1, UPP2*, *CCDC148*, and *PKP4*).

Various in silico tools predicted that the variant in patient 2 affects splicing that would lead to LoF. This was confirmed by RT-PCR (see Supplementary Figure S2), which revealed altered splicing leading to aberrant transcripts with an out of frame skipping of exon 4 (130 nucleotides), which will potentially cause truncation and LoF through NMD.

All variants had high predictive scores for a detrimental effect as predicted by SIFT, PolyPhen-2, and CADD scores. The *NR4A2* gene appears to be under a high selective strain and extremely intolerant to LoF variation as evidenced from its high probability of being LoF intolerant (pLI) score (1.0) and lower than expected missense variant counts (*Z*-score = 2.24), as observed in both ExAC and gnomAD. The haploinsufficiency score (HI score = 1.28%) shows this gene to be highly dosage sensitive. Therefore, intolerance to LoF can play an important role in the development of pathogenic phenotypes in patients.^3,10,11^

## DISCUSSION

We report nine patients with early onset epilepsy and/or a developmental disorder, of whom eight carried intragenic variants and on a larger deletion including *NR4A2*. Six of these patients with de novo *NR4A2* variants had epilepsy. The apparent intolerance to LoF and missense variation of *NR4A2* suggests that these variants are causing the phenotype in patients.^3,10,11^ In previous studies, haploinsufficiency of *NR4A2* has been implicated in a neurodevelopmental phenotype, including significant language impairment^11,12^ and ID.^3,7^ These symptoms overlap with those observed in the patients studied here. We also observed language impairment in five of nine patients, which may be linked to the more prominent expression of *NR4A2* in the superior temporal gyrus (STG), a brain region linked to language development.^13^

Regardless of the similarities among these nine patients, the underlying explanations for the phenotypes of patient 4 and patient 9 may be different. Patient 4 had a deceased sibling with similar phenotype. Therefore, the de novo variant detected in patient 4 may not explain the full phenotype, and another cause, as well as germline mosaicism, should be considered. For patient 9 the microdeletion also affected ten other genes that could have contributed to the clinical features. For example, *KCNJ3*, also deleted in this patient, encodes for subunit G-protein activated inward rectifier potassium channel 1. Alterations in the function of this potassium channel subunit have been associated with epilepsy.^14^

It remains unclear how these variants of *NR4A2* contribute to the epileptogenesis. However, the physiologic role of *NR4A2* provides a clue toward the complex phenotypes of the patients with variants in this gene. NR4A2 is a known transcription factor and it binds to DNA as monomer or homodimer to promote constitutive activation of transcription.^15^ It exerts concentration-dependent effects on target genes mediating distinct biological processes.^16^ The product of a mutated gene can be subjected to NMD leading to a misfolded protein, or a dysfunctional ligand binding pocket, or a dysfunctional DNA binding, resulting in impaired or loss of function. It is important to take into consideration that amino acid substitutions can also disrupt the conformation of the protein and such conformational changes might also compromise the function of *NR4A2*. A large case–control study of patients with neurodevelopmental disorders (such as autism spectrum disorder, DD, ID, and epilepsy) showed significant clustering of the de novo missense variants in cases at the protein level for 200 genes including *NR4A2*. In many cases, these de novo variants clustered in protein functional domains (such as zinc finger motifs, transmembrane domains, voltage sensors, and channel pores) relevant in the neurodevelopmental pathology. The de novo variants in zinc finger motifs were not present in public control databases. A clustering of variants in specific functional domains emphasized the importance of these de novo variants in characterizing pathogenic genes and functional domains.^17^ The DBD of *NR4A2* contains two highly conserved C4 type zinc fingers that bind to specific motifs in DNA hormone response elements. Two of six patients with epilepsy have a missense variant in the DBD, whereas one patient has a missense variant in a hinge region with no secondary structure in the LBD and in N-terminal regulatory region of the protein. The two missense variants in the highly conserved DBD of NR4A2 may have resulted in impaired DNA binding ability. The other variants observed in patients are truncating variants and deletions, which taken together with the observed clustering of missense variants in a biological relevant protein domain strongly suggest that LoF is the underlying disease mechanism.

It is well established that *NR4A2* is important for differentiation, survival and maintenance of dopaminergic neurons.^1^ Furthermore, the dopamine activity transporter *DAT* gene has been associated with idiopathic absence epilepsy and seizures.^18^ Various animal studies have investigated how the DAergic system is linked to epileptogenesis and neurodevelopmental dysfunction.^1^ Different subtypes of the dopamine receptors can act either as proconvulsant (D1-like receptor) or anticonvulsant (D2-like receptor). Physiological balance of DAergic activity at D1R and D2R can be decisive for complex neuromodulatory response for epileptogenesis.^16^ The knockout or knockdown of *NR4A2* have resulted in poor motor function and lower number of DA neurons, lower levels of protein, and reduced DA at birth in midbrain.^19^ Studies of *NR4A2* heterozygosity in animal models have shown that the affected dopaminergic system is associated with altered locomotor behavior.^20^ Furthermore, a crucial role of *NR4A2* in overall survival was supported by animal studies revealing its involvement in respiratory abnormality and lack of response to hypoxia. *NR4A2* is encoded by immediate early genes and has a significant role in development, neuroprotection, learning, and memory formation,^19^ presenting a reasonable explanation for the associated ID among these patients. These studies indicate that variants in *NR4A2* can impair its various functions, and plausibly contribute to the seizure, neurodevelopmental, and global developmental phenotype observed in the described patients.

## Supplementary information

Supplementary Information
