# Supplementary material for: De novo variants of NR4A2 are associated with neurodevelopmental disorder and epilepsy
Source: Genet Med. 2020 May 5;22(8):1413–7. doi: 10.1038/s41436-020-0815-4 (PMC7394879; doi:10.1038/s41436-020-0815-4)

**Supplementary Information**

***De novo* variants of *NR4A2* are associated with neurodevelopmental disorder and epilepsy**

^1^Sakshi Singh PhD, ^2,3^Aditi Gupta PhD, ^4,5^Michael Zech MD, ^2,6^Ashley N. Sigafoos BS, ^2,6^Karl J. Clark PhD, ^7,8^Yasemin Dincer MS, ^4,5^Matias Wagner MD, ^9^Jennifer B. Humberson MD, ^10^Sarah Green MS, ^1^Koen van Gassen PhD, ^11^Tracy Brandt PhD FACMG, ^11^Rhonda E. Schnur MD FACMG, ^11^Francisca Millan MD FACMG, ^11^Yue Si MD Ph.D, ^7,12^Volker Mall MD, ^4,5,13,14^Juliane Winkelmann MD, ^2,15,16^Ralitza H. Gavrilova MD, ^2,3,15^Eric W. Klee PhD, ^17^Kendra Engleman MS, ^17^Nicole P. Safina MD, ^18^Rachel Slaugh MS, ^19^Emily M. Bryant MS, ^20^Wen-Hann Tan BMBS, ^18^Jorge Granadillo MD, ^19^Sunita N. Misra MD PhD, ^10^G. Bradley Schaefer MD, ^9^Shelley Towner MS, ^1^Eva H. Brilstra MD PhD, ^1^Bobby P. C. Koeleman PhD

Affiliations:

^1^Department of Genetics, University Medical Centre Utrecht, The Netherlands

^2^Center for Individualized Medicine, Mayo Clinic, Rochester, Minnesota, USA

^3^Department of Health Sciences Research, Mayo Clinic, Rochester, Minnesota, USA

^4^Institut für Neurogenomik, Helmholtz Zentrum München, Munich, Germany

^5^Institut für Humangenetik, Klinikum rechts der Isar, Technische Universität München, Munich, Germany

^6^Department of Biochemistry and Molecular Biology, Mayo Clinic, Rochester, Minnesota, USA

^7^Lehrstuhl für Sozialpädiatrie, Technische Universität München, Munich, Germany

^8^Zentrum für Humangenetik und Laboratoriumsdiagnostik (MVZ), Martinsried, Germany

^9^Department of Pediatrics, University of Virginia, Charlottesville, Virginia, USA

^10^Section of Genetics and Metabolism, University of Arkansas for Medical Sciences, Little Rock, Arkansas, USA

^11^GeneDx, Gaithersburg, Maryland, USA

^12^kbo-Kinderzentrum München, Munich, Germany

^13^Lehrstuhl für Neurogenetik, Technische Universität München, Munich, Germany

^14^Munich Cluster for Systems Neurology, SyNergy, Munich, Germany

^15^Departments of Clinical Genomics and Neurology, Mayo Clinic, Rochester, Minnesota, USA

^16^Department of Neurology, Mayo Clinic, Rochester, Minnesota, USA

^17^Division of Clinical Genetics, Children’s Mercy Kansas City, University of Missouri Kansas City School of Medicine, USA

^18^Division of Genetics and Genomic Medicine, Department of Pediatrics, Washington University School of Medicine, St. Louis, Missouri, USA

^19^Ann & Robert H. Lurie Children's Hospital, Epilepsy Center, Chicago, Illinois, USA

^20^Division of Genetics and Genomics, Department of Medicine, Boston Children’s Hospital, Harvard Medical School, Boston, Massachusetts, USA

***NR4A2* Sequencing methods.**

Patient 3 was identified as part of a research project to identify genes associated with dystonia and other hyperkinetic disorders. Variants in patient 1, 6, and 9 were discovered in clinical diagnostic settings. For patients 1, 3, and 9, genomic DNA was extracted from peripheral blood from the affected patients and their family members by standard procedures.

GeneDx patients were sequenced using massively parallel (next generation) sequencing was done on an Illumina system with 100 bp or greater paired-end reads. Reads were aligned to human genome build GRCh37/UCSC hg19, and analysed for sequence variants using a custom-developed analysis tool. Additional sequencing technology and variant interpretation protocol has been previously described^13^.

***NR4A2* RT-PCR Methods**

RNA samples were obtained and stored at -80^o^C. RNA sample was synthesized into cDNA via Invitrogen’s SuperScript™ II Reverse Transcriptase (cat. 18064-014) using random hexamers. Once cDNA was synthesized, MyTaq™ DNA Polymerase (Bioline cat. BIO-21105) PCR kit was used to amplify the region around exon 4. Primers were designed and purchased from Integrated DNA Technologies, LLC (Table S1) targeting exon 4 region to identify potential splice acceptor alteration. Both proband cDNA and control cDNA samples were amplified using RT-NR4A2-F1/R1 primer set, which should produce an estimated 679bp fragment. PCR product was run on a 1% agarose gel (Figure S1). Two fragments were observed in the proband sample with the expected 679bp wildtype fragment and then a smaller product around 580bp. All three fragments (control 679bp, proband 679bp, and proband 580bp) were excised from the gel and gel purified (QIAquick Gel Extraction Kit, QIAGEN cat: 28704), and TOPO™ cloned (TOPO ™ TA Cloning™ Kit, Thermo cat: 450641). White colonies were selected and grown in culture and DNA was isolated using QIAperep^®^ Spin Miniprep Kit (QIAGEN, cat: 27106). Sanger sequencing was performed on the plasmids using primers provided by the TOPO™ cloning kit (M13 Forward) to identify effect of intronic mutation. Twelve colonies from each of the three fragments were sequenced and it was shown that in the 679bp fragment in both control cDNA and proband cDNA produced the wildtype sequence of *NR4A2*, while the smaller fragment of 580bp in the proband confirmed aberrant transcript produced by mis-splicing due to the splice variant (Figure S2).

***NR4A2* RT-PCR Results**

The *NR4A2* variant c.865-1_865delGCins10, was present in intron 3 of an 8 exon transcript (NM_006186.3). Various *in silico* tools like MaxEntScan, NNSPLICE, Human Splicing Factor predicted this variant to affect splicing and potentially lead to loss-of-function (LoF). This was confirmed by RT-PCR, which revealed altered splicing leading to aberrant transcripts with an out of frame skipping of exon 4 (130 nucleotides), which will potentially cause truncation, hence loss of function of protein. This gene is predicted to be intolerant to LoF and missense variants on ExAC Browser.

**Analysis of pathogenic enriched regions using the *NR4A2* gene family**

The *NR4A2* gene belongs to a gene family *NR3C1*, *NR3C2*, *PGR*, *AR*, *NR4A1*, and *NR4A3*. The Pathogenic Enriched Region PER analysis compares the rate of missense variation observed in the general population to that reported for patients to identify gene regions that are enriched for patient variations (http://per.broadinstitute.org/; Perez-Palma et al. Genome Research 2020;30:62-71). When applying this method to NR4A2, we observed 11 PER’s (Figure S4). All missense variants observed in our patients were located in a PER, further supporting their pathogenicity.

**Patient phenotype description**

*Patient 1:* The patient is the child of two unrelated parents. She was 15 years old at her last visit to the clinic. Her father had absence epilepsy in childhood. She had normal development until the age of 18 months when she had a febrile illness with global DD thereafter. She started having tonic seizures at the age of 6.5 years which occurred more than 10 times a day. She additionally developed tonic-clonic seizures when she was 8 years old. These seizures were characterized by impaired awareness and left hemiparesis. She had been treated with carbamazepine and valproate, later with clonazepam and sulthiame. She responded well to clonazepam and sulthiame and became seizure free at the age of 11.5 years. She has severe ID, autism and behavioural problems with tantrums and aggression, and sleeping difficulties. Brain MRI was normal. EEG (electroencephalogram) showed epileptic discharges in the central temporal regions at the onset and later on also generalized epileptic discharges.

*Patient 2:* The patient is the child of two unrelated parents. The father and the maternal grandfather of the patient had mild learning disability. A maternal uncle had adult-onset generalized tonic-clonic seizures. Diagnosis of benign rolandic epilepsy was made at the age of 10 years. The patient displayed bilateral extremity twitching and, head shaking which usually occurred at night. These symptoms were also associated with enuresis and staring episodes. He responded well to initial treatment with oxcarbamazepine (Trileptal) and became seizure free. The patient was 12 years old at the time of his last visit, and had mild cognitive impairment with an IQ of 72, speech apraxia, and dyslexia. His MRI findings were normal. The video EEG showed frequent right and left centrotemporal epileptiform discharges with associated dipole, which are sleep activated. This may represent underlying regions of cortical irritability over bilateral centrotemporal regions with lowered seizure threshold. He had mild hypotonia, headaches, and an abnormal awake and sleep routine. The patient also showed hyperactivity, anxiety and unusual behaviour. Other associated features included cryptorchidism, mild scoliosis, easy fatigability, mild muscle weakness, chronic constipation, mild myopia, mild skin hyperextensibility and joint hypermobility in the setting of hypotonia.

*Patient 3*: The patient is the child of two unrelated healthy parents with no family history of epilepsy. She was 9 years old at the time of her last visit and then had mild to moderate ID. She had no epileptic seizures but her EEG showed epileptiform abnormalities and photosensitivity. She is treated with oxcarbazepine. She had movement disorders, including choreo-athetoid movements, dystonia and ataxia. Her MRI showed abnormal signal intensities indicating gliosis of the lateral thalamus and the dorsal basal ganglia.

*Patient 4*: The patient is the child of two unrelated healthy parents with no family history of epilepsy. Her deceased sister reportedly had a similar phenotype, but was not genetically tested. The disease started with infantile spasms at the age of 5 months which further developed to a severe generalized encephalopathy characterized by global DD, dystonia, infantile seizures with hypsarrhythmia, dystonia and microcephaly. She exhibited severe ID. Her MRI showed moderate cerebellar atrophy and foci of hemosiderin deposition (Supplementary Figure S3). Her EEG showed bilateral temporal lobe spikes at the time of seizure onset, and a generalized slowing at the age of 3.5 years. Her neurological symptoms were severe hypotonia, dystonia and cerebral visual impairment. The patient had an excellent response to vigabatrin (Sabril) treatment, which resulted in seizure freedom after the first year of age. The patient was 3 years old at the time of her last visit and at that time the decision was to wean her off of vigabatrin treatment soon.

*Patient 5*: The patient is the child of two presumed unrelated parents, but the biological father was not available for investigation. His mother was diagnosed with anxiety and severe drug abuse. She was using drugs during pregnancy. The maternal family history was positive for schizophrenia. The patient was 7 years old at the time of his last visit and showed global DD, receptive-expressive language delay, fine motor delay, sensory processing disorder and speech apraxia. He showed symptoms of attachment disorder, most likely due to a history of neglect, and of attention deficit hyperactivity disorder (ADHD). Other abnormalities included hyposensitivity to temperatures and pain, photosensitivity, mild hypotonia, cerebral visual impairment, astigmatism and exotropia. Dysmorphic facial features were a slightly prominent nasal bridge and deep set eyes.

*Patient 6*: The patient is the first child of healthy unrelated parents. He was IUGR at birth and had hyperbilirubinemia at 3 days of life treated with phototherapy. At 3 months of age, his parents became concerned about poor development and noted abnormal eye movements. At 6 months, he showed shaking eye movements that correlated with right temporal sharp waves on EEG, and he was placed on levetiracetam for focal seizures. He developed infantile spasms around 10-11 months of age and was treated with prednisolone. His brain MRI showed pontine hypoplasia, ventricuolomegaly and delayed myelination around 16 months of age. He subsequently has evolved into Lennox-Gastaut syndrome with frequent tonic and myoclonic-tonic seizures that have been refractory to numerous medications and the ketogenic diet. He has global DD and receives physical, occupation, speech, development, and vision therapies. He is G-tube dependent, has severe hypotonia, and sleep disordered breathing.

*Patient 7*: Patient 7 is a female child of unrelated parents born to a mother who had a history of FV Leiden treated with heparin during pregnancy. She had normal development until the parents noticed a delay in motor and language development and short attention span at 12 months of age. She was noted to have global DD, hypotonia, and a shagreen patch. She was enrolled in a child special education setting. She speaks in up to 5-word sentences, and was able to walk at 16 months of age. Metabolic screening and brain MRI were normal. The patient had no history of seizures.

*Patient 8*: The patient is a female child of unrelated parents with a negative family history. She presented with global DD between 8 months and 1 year of age. She was able to walk, but not run or ride a bicycle, at 4 years of age. She has severe language impairment and was diagnosed as having moderate to severe intellectual impairment. Her language skills were disproportionately delayed compared to her motor skills and overall cognition. The patient had normal brain MRI and no history of seizures.

*Patient 9:* The patient is the first child of healthy unrelated parents. He had normal early development. At age 2.5 years his development stagnated and he started to show severe behavioural problems, including aggression and destructive behaviour. At the age of 13 years epilepsy was diagnosed. MRI showed enlarged cerebrospinal fluid spaces. He had absence seizures and later on also developed tonic seizures, focal seizures with impaired awareness and drop attacks, which were refractory to treatment. He has a moderate ID and progressive ataxia as an adult.

**MRI finding**

MRI of patient 4 shows moderate cerebellar atrophy and foci of likely hemosiderin (Figure S3).

**Table S1:** RT-PCR primer information for patient 2

| Primer Name | Sequence 5’-3’ |
| --- | --- |
| RT-NR4A2-F1 | gagcagaggaaaacgccagt |
| RT-NR4A2-R1 | tcgcctggaacctggaatagtc |
| M13-Forward | gtaaaacgacggccag |


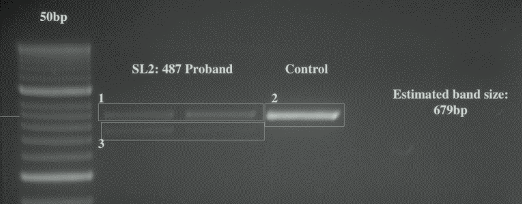


**Figure S1:** RT-PCR gel run for patient 2

Figure shows agarose gel containing the products of the RT-PCR on NR4A2 products. Lane 2 and 3 shows the product obtained from patient 2, lane 4 of a control sample. The control and patient samples shows only a product of expected size of 679bp (bands 1 and 2), whereas an aberrant smaller product (band 3) is visible only in the patient samples.


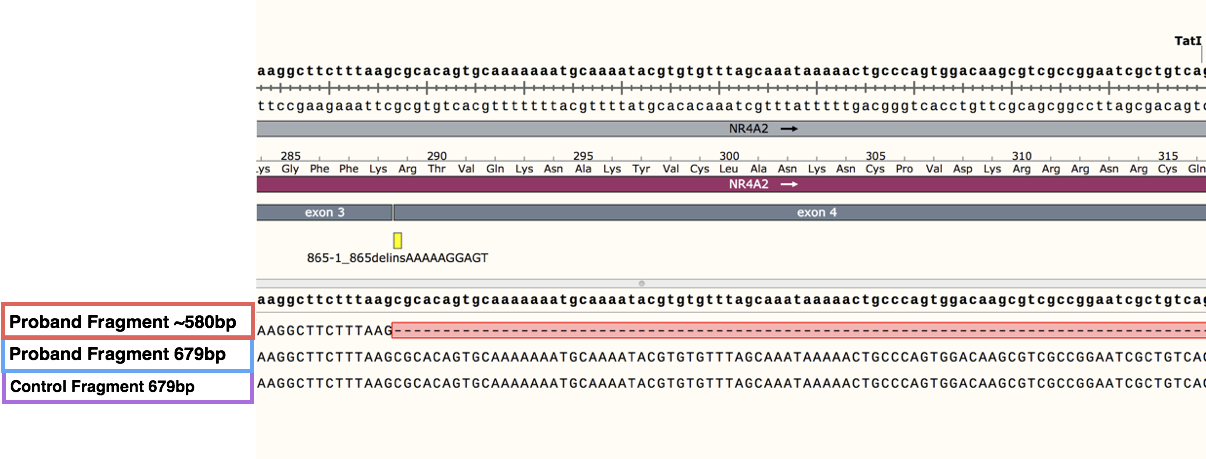


**Figure S2:** Splice variant sequence in patient 2.

Figure shows the cDNA sequence of the normal NR4A2 product (genomic reference sequence on top, cDNA reference sequence fourth line from the bottom) and for the two expected alleles of the patient carrying the 86-1-865delinsAAAAAGGAGT deletion (third and second line from the bottom, .

**Figure S3:** Midsagittal MRI image of patient 4.

***
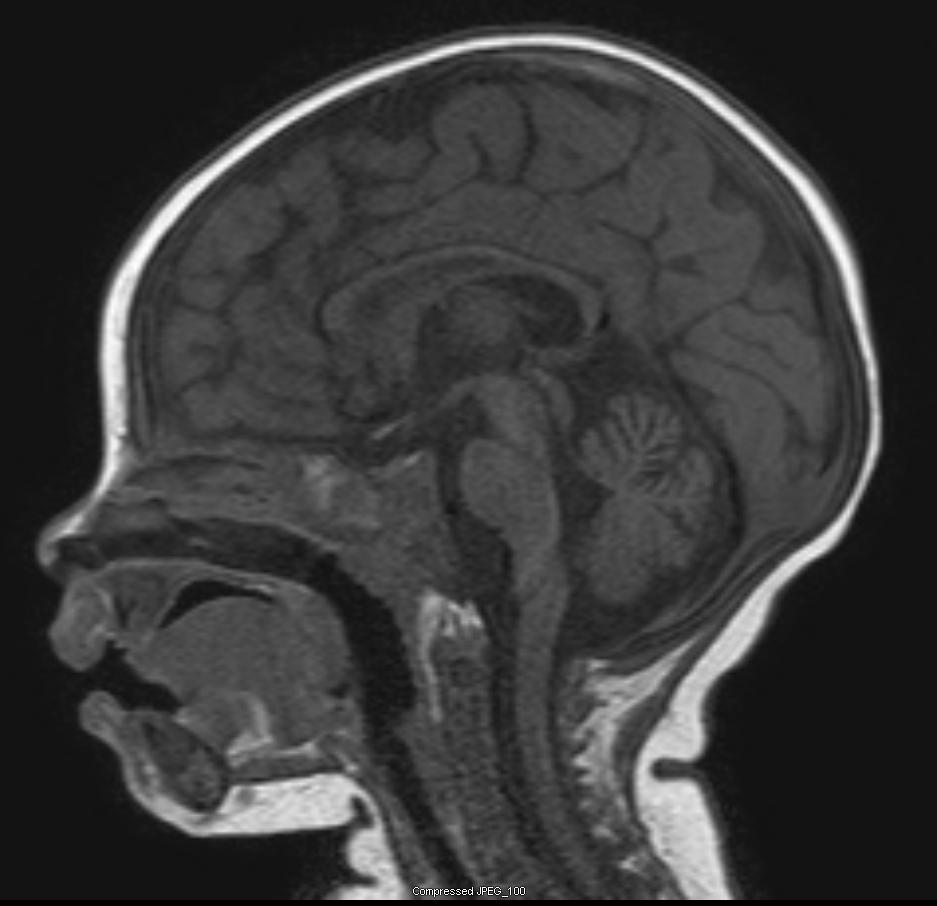
***

**Figure S4:** Analysis of missense pathogenic enriched regions of the NR4A2 gene family.


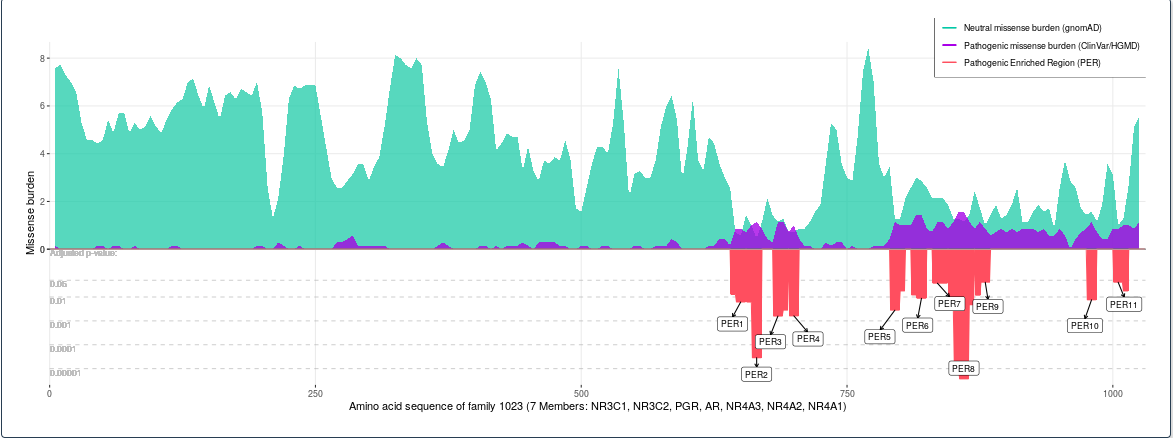

Supplement: Supplementary file 1 — Supplementary Information [file 41436_2020_815_MOESM1_ESM.docx]
